# Performance of deepseek-R1 and ChatGPT-5.4 thinking in the medical laboratory professional title examination: accuracy, stability, and comparison with interns

**DOI:** 10.3389/fdgth.2026.1858993

**Published:** 2026-06-19

**Authors:** Zhili Niu, Dongling Tang, Juanjuan Chen, Pingan Zhang, Chengliang Zhu

**Affiliations:** 1 Department of Clinical Laboratory, Renmin Hospital of Wuhan University, Wuhan, China; 2 Institute of Clinical Molecular Diagnostics, Wuhan University, Wuhan, China; 3 Hubei International Science and Technology Cooperation Base for Digital and Intelligent Molecular Diagnostics, Wuhan, China

**Keywords:** artificial intelligence, ChatGPT-5.4 thinking, DeepSeek-R1, interns, medical laboratory, professional title examination

## Abstract

**Objective:**

To systematically evaluate the accuracy, reproducibility, and performance of Deepseek-R1 and ChatGPT-5.4 Thinking across different question types and disciplines in the Medical Laboratory Junior Professional Title Examination, and to compare their performance with that of interns.

**Methods:**

Four examination papers comprising a total of 3,879 questions were independently administered to both models in three repeated sessions. Accuracy rates were recorded, and reproducibility was assessed. Performance was further compared across three question types and five disciplines. In addition, 46 final-year interns were recruited, and their accuracy rates were compared with those of the two models.

**Results:**

Neither model showed significant differences in accuracy across the three repeated sessions (*p* > 0.05), and both demonstrated good reproducibility and stability, with Fleiss' kappa coefficients exceeding 0.7 (*p* < 0.001). No significant differences in accuracy were observed across question types for either model (*p* > 0.05). Across disciplines, Deepseek-R1 showed no significant differences across disciplines (*p* > 0.05), whereas ChatGPT-5.4 Thinking exhibited significant cross-disciplinary differences in Papers I, II, and III (*p* < 0.05). Inter-model comparison revealed that Deepseek-R1 achieved significantly higher accuracy than ChatGPT-5.4 Thinking in Papers I, II, and III (*p* < 0.05), while no significant difference was observed in Paper IV (*p* > 0.05). Both models achieved higher accuracy than interns on most papers; interns performed comparably to the AI models on Paper I but scored substantially lower on Papers II, III, and IV. Deepseek-R1 showed the highest overall performance. Analysis of error types indicated that the highest proportion of errors were those consistently incorrect across all three repetitions, suggesting stable knowledge gaps.

**Conclusion:**

Both Deepseek-R1 and ChatGPT-5.4 Thinking demonstrated strong performance and reproducibility in the Medical Laboratory Junior Professional Title Examination. Deepseek-R1 showed superior overall accuracy and greater disciplinary consistency. Both models outperformed fourth-year interns, highlighting their potential as auxiliary tools for examination preparation, though the use of publicly available historical questions limits conclusions about genuine reasoning ability.

## 1 Introduction

Recent advances in artificial intelligence have had a profound impact on medical education (1). Generative AI, particularly large language models, is now playing a significant role in this field. It is reshaping the way knowledge is delivered, while also influencing how clinical reasoning is taught and how standardized examinations are evaluated (2–4). In medical education, AI can provide personalized learning support for students. It also helps educators design teaching cases and simulate clinical scenarios, ultimately contributing to improved educational outcomes (5). As AI models become more capable, their performance on medical exams serves as a key benchmark for assessing their medical knowledge and clinical reasoning skills.

Among the various large language models, the ChatGPT series stands out as one of the most influential. Since its launch in late 2022, ChatGPT has experienced exponential growth, reaching 400 million weekly active users by 2025 (6). ChatGPT-4o, a free multimodal model capable of processing text, images, and audio with rapid response times and strong multilingual support, has demonstrated performance comparable to or even exceeding that of human examinees in several medical exams (7). For instance, it achieved passing scores in the Polish Medical Graduation Examination and Dental Examination, performed well in the United States Medical Licensing Examination Step 1 and Step 2, and exhibited stable reasoning capabilities in specialty exams such as cardiology and dermatology (6). In January 2025, DeepSeek-R1, developed by the Chinese company DeepSeek, emerged as a high-performance, low-cost, and open-source alternative (8, 9). The model was trained at a cost of only $6 million, substantially lower than the reported $100 million for OpenAI's GPT-4, and required only one-tenth of the computational resources (10). DeepSeek-R1 achieved reasoning capabilities comparable to ChatGPT-o1 in tasks involving mathematics, programming, and scientific reasoning. It has been specifically optimized for Chinese-language contexts, which enhances its adaptability in handling Chinese medical questions. Within a week of its release, its mobile application surpassed ChatGPT in downloads on the U.S. App Store, reflecting strong user interest (8, 9). Studies have shown that DeepSeek-R1 outperformed ChatGPT in knowledge-recall tasks on USMLE Step 1 and Step 2, and in complex clinical case analyses on Step 3, its reasoning steps were closer to correct answers than those of OpenAI's models (6).

To date, numerous studies have evaluated the performance of large language models in various medical examinations. GPT-4, for instance, achieved an accuracy of 84.9% on the Chinese National Medical Licensing Examination (11). Recent research has increasingly focused on comparative performance across different models. Tordjman M et al. evaluated Deepseek, ChatGPT, and Llama 3.1 on healthcare tasks and found that DeepSeek provided more accurate diagnostic reasoning steps (12). Ming, S. et al. found that GPT-4o surpassed GPT-3.5 in accuracy, consistency, and subspecialty knowledge on the Chinese Medical Licensing Examination (13). Similarly, Tassoker, M. et al. compared ChatGPT-3.5, GPT-4o, Google Bard, and Microsoft Copilot on oral radiology examinations, confirming the superior accuracy and reasoning capabilities of GPT-4o (14). Although existing studies have conducted multi-model comparisons, they have largely focused on fields such as clinical medicine, pathology, and oral medicine, with limited attention paid to medical laboratory technology. Furthermore, most studies have relied on earlier model versions, such as GPT-4, with limited evidence available for newer models such as ChatGPT-5.4. To date, no study has systematically investigated the performance of different large language models in the Chinese Medical Laboratory Technologist Qualification Examination. This examination is a critical component of China's health professional qualification system. It covers foundational medicine, clinical laboratory techniques, and laboratory quality management, and demands not only solid theoretical knowledge but also advanced clinical judgment.

Given DeepSeek's strength in Chinese-language contexts and ChatGPT's strength in multimodal processing, it is essential to compare these two models in this specialized exam. Such a comparison can reveal their suitability for domain-specific tasks and provide evidence to guide the informed selection of AI tools in medical education. Therefore, this study aims to systematically assess and compare the performance of Deepseek-R1 and ChatGPT-5.4 in the Chinese Medical Laboratory Technologist Qualification Examination. The analysis focuses on their differences in accuracy, consistency, and overall answer stability. The goal is to fill this research gap and offer both theoretical support and practical guidance for the deeper integration of AI into medical education.

## 2 Methods

### 2.1 Study materials

#### 2.1.1 Source of test questions

This study collected historical examination papers from the Medical Laboratory Junior Professional Title Examinations spanning a decade from 2015 to 2024. The examination consists of four subjects: Basic Knowledge (I), Related Professional Knowledge (II), Professional Knowledge (III), and Professional Practice Ability (IV). These subjects form a hierarchical assessment system constructed based on the competency requirements for professionals in clinical laboratory positions. They systematically evaluate laboratory professionals' expertise and job competence from four dimensions: theoretical foundation, interdisciplinary knowledge and quality standards, core professional techniques, and clinical practice with problem-solving abilities.

Each of the four examination papers originally contained 1,000 questions. The number of image-based questions was very small (Paper I: 28; Paper II: 9; Paper III: 11; Paper IV: 43). The electronic version of the examination materials we purchased contained only black-and-white images with poor resolution and low contrast, which made accurate interpretation unreliable. Because the small number of image-based questions precluded any meaningful stratified statistical analysis, and the low image quality further compromised reliability, all image-based questions were excluded from this study. After exclusion, the numbers of questions included for analysis were: 972 in Paper I, 991 in Paper II, 989 in Paper III, and 957 in Paper IV, giving a total of 3,879 questions.

To enable in-depth analysis of model performance across different question types and disciplinary areas, all questions were categorized by format into three types: clinical knowledge questions, theoretical knowledge questions, and case analysis questions. By content, they were classified into five disciplines: Clinical Laboratory Fundamentals, Immunology, Microbiology, Biochemistry, and Hematology. Each question was accompanied by officially published standard answers and detailed explanations, which served as the reference standard for evaluating model outputs. Two medical laboratory technologists with intermediate professional titles independently verified all standard answers and explanations; any discrepancies were resolved by a third expert with a senior professional title.

#### 2.1.2 Models and testing procedures

Two mainstream large language models were selected for comparison: Deepseek-R1 and ChatGPT-5.4 Thinking. Deepseek-R1, developed by DeepSeek AI, has knowledge updated to May 2025. It represents a further optimization based on DeepSeek-R1, with deep tuning for Chinese medical contexts and enhanced reasoning capabilities at a lower cost. ChatGPT-5.4 Thinking, developed by OpenAI, has training knowledge updated to August 2025. As the latest iteration in the ChatGPT series, it features significant improvements in multimodal processing, complex reasoning, and response stability, representing the current international state-of-the-art. Both models were tested with their thinking mode enabled.

To assess model stability, each paper was administered to both models three times, with at least 24 h between sessions. To prevent any memory leakage across test sessions, we started each test session with a completely new conversation thread, giving the models no recollection of previous answers. For ChatGPT, we also turned off its persistent memory feature in the settings (15). DeepSeek needed no such adjustment—its web-based chat interface has no persistent memory by default (https://deepseek.international/deepseek-chat-memory-and-context-length-explained/). Finally, we confirmed that web search was disabled for both models throughout the testing.

A standardized prompt template was used for both models: “You are a professional medical laboratory technologist. Please select the correct option for the medical laboratory professional title examination questions I provide and provide an answer explanation.” Both models received identical prompts to ensure fair comparison.

All tests were conducted through the official web-based chat interfaces of DeepSeek and ChatGPT, rather than via their respective APIs. For each test session, a new conversation thread was initiated without any prior context or memory. The prompts were entered manually by the researchers, and the models' generated responses were recorded directly from the chat interface.

#### 2.1.3 Evaluation metrics

##### 2.1.3.1 Accuracy

Accuracy was defined as the percentage of correctly answered questions out of the total questions attempted. Accuracy rates were calculated for both models across the four papers, five disciplinary classifications, and three question-type classifications, and differences between the two models were compared. The official passing score for each of the four examination papers is 60 out of 100 (i.e., 60% accuracy). National passing rates or mean scores for this examination are not publicly released; therefore, we used the 60% threshold as the only external benchmark.

##### 2.1.3.2 Consistency

Consistency reflected the stability of model outputs across repeated tests. For each question, if the model selected the same option in all three independent sessions, the response was considered perfectly consistent. The Fleiss' Kappa coefficient was used to quantify overall consistency across the three sessions.

##### 2.1.3.3 Intern test scores

Forty-six final-year medical laboratory undergraduates participated in the study. Inclusion criteria were: 1) full-time undergraduate students enrolled in Medical Laboratory Technology programs; 2) completion of three years of systematic professional coursework, including core courses such as Clinical Laboratory Fundamentals, Hematology, and Biochemistry; and 3) completion of 10 months of clinical rotations across different sections within the clinical laboratory department.

They were from two universities, Yangtze University and Hainan Medical University, and had completed their 10-month required clinical rotations in our department prior to the test. 22 of them were from Yangtze University and 24 from Hainan Medical University. The test questions consisted of the four papers (I–IV) from the 2024 Medical Laboratory Junior Professional Title Examination. The interns completed each paper under strictly closed-book conditions, without access to any reference materials, electronic devices, or internet resources, following the standard examination regulations for the Medical Laboratory Junior Professional Title Examination. Each paper was allocated 90 min, consistent with the official examination duration for this qualification. The time taken by each intern to complete each paper was recorded, and all interns finished within the allotted time. The interns completed these tests in March 2026.

For the AI-intern comparison, only the 2024 examination papers were used to calculate the mean accuracy of the two AI models across three repeated sessions.

### 2.2 Statistical analysis

All statistical analyses were performed using SPSS version 27.0. Categorical data were presented as frequencies and percentages, and comparisons between groups were conducted using chi-square tests. Cochran's *Q-*test was used for repeated-measures comparisons of categorical accuracy data across the three testing sessions, while chi-square tests were adopted for between-group comparisons of independent categorical data. For non-normally distributed data, the Mann–Whitney *U*-test was used for multiple group comparisons. Accuracy comparisons between the two models were performed using McNemar's test (paired design), and the corresponding discordant pair counts were reported. Consistency across the three testing sessions was assessed using Fleiss' Kappa coefficient, with Kappa values interpreted as: <0.20 poor, 0.21–0.40 fair, 0.41–0.60 moderate, 0.61–0.80 substantial, and 0.81–1.00 almost perfect agreement. The significance level was set at *α* = 0.05, with *P* < 0.05 considered statistically significant. To characterize the stability of model errors, incorrect answers across the three repeated sessions were categorized into three patterns: incorrect in all three sessions, incorrect in exactly two sessions, and incorrect in exactly one session. The distribution of these patterns was described across question types and disciplines. No inferential statistics were applied to this descriptive analysis.

## 3 Results

### 3.1 Repeatability validation of AI model accuracy

To assess the stability of the AI models, both Deepseek-R1 and ChatGPT-5.4 Thinking completed four test papers (I–IV) in three separate sessions. The consistency of their accuracy rates was then compared. The results showed no statistically significant differences in accuracy across the three test administrations for either model on any of the paper types (*p* > 0.05), indicating good repeatability and stability of both models in terms of answering accuracy (Table 1).

**Table 1:** Reproducibility of accuracy rates for two AI models across four examination.

Paper Type	Repetition	Deepseek-R1		ChatGPT-5.4 Thinking	
		Correct	Wrong	Correct	Wrong
I Basic Knowledge (*n* = 972)	1st	906 (93.21)	66 (6.79)	871 (89.61)	101 (10.39)
	2nd	906 (93.21)	66 (6.79)	869 (89.40)	103 (10.60)
	3rd	909 (93.52)	63 (6.48)	874 (89.92)	98 (10.08)
	Cochran's Q	0.241		0.731	
	*P*	0.886		0.694	
II Related Professional Knowledge (*n* = 991)	1st	903 (91.12)	88 (8.88)	862 (86.98)	129 (13.02)
	2nd	907 (91.52)	84 (8.48)	858 (86.58)	133 (13.42)
	3rd	904 (91.22)	87 (8.78)	854 (86.18)	137 (13.82)
	Cochran's Q	0.121		0.300	
	P	0.941		0.861	
III Professional Knowledge (*n* = 989)	1st	918 (92.82)	71 (7.18)	888 (89.79)	101 (10.21)
	2nd	922 (93.23)	67 (6.77)	885 (89.48)	104 (10.52)
	3rd	923 (93.33)	66 (6.67)	881 (89.08)	108 (10.92)
	Cochran's Q	0.230		0.279	
	*P*	0.892		0.870	
IV Professional Practice Ability (*n* = 957)	1st	869 (90.80)	88 (9.20)	848 (88.61)	109 (11.39)
	2nd	872 (91.12)	85 (8.88)	845 (88.30)	112 (11.70)
	3rd	873 (91.22)	84 (8.78)	833 (87.04)	124 (12.96)
	Cochran's Q	0.116		1.36	
	P	0.944		0.507	

Data are presented as *n* (%). Percentages represent the proportion of correct or incorrect answers within each repetition.

### 3.2 Consistency analysis across three repeated administrations

To evaluate the stability of AI model responses across repeated administrations, the consistency of answers generated by the two large language models across three test rounds was analyzed. The results showed that both Deepseek-R1 and ChatGPT-5.4 Thinking demonstrated high consistency across different types of medical examination papers. All Fleiss' kappa coefficients were greater than 0.7 (*p* < 0.001), indicating good response reproducibility for both models across multiple test administrations. The detailed results are presented in Table 2.

**Table 2:** Consistency of responses across three repeated administrations.

Paper type	Deepseek-R1			ChatGPT-5.4 Thinking		
	Fleiss' kappa coefficient	Z-score	*p* value	Fleiss' kappa coefficient	Z-score	*p* value
I Basic Knowledge	0.724	39.09	<0.001	0.808	43.63	<0.001
II Related Professional Knowledge	0.784	42.76	<0.001	0.754	41.11	<0.001
III Professional Knowledge	0.753	40.99	<0.001	0.806	43.17	<0.001
IV Professional Practice Ability	0.842	45.11	<0.001	0.764	41.63	<0.001

Fleiss' kappa coefficient was used to assess inter-rater agreement across three sessions. A kappa value of <0.2 indicates slight agreement; 0.2–0.4, fair; 0.4–0.6, moderate; 0.6–0.8, substantial; and 0.8–1.0, almost perfect agreement.

### 3.3 Analysis of AI model accuracy across different question types

Given the high consistency across the three sessions, the results from the first session were used for further analysis of accuracy across different question types (clinical knowledge, basic theory, and case analysis). The results showed that, across the four test papers, no statistically significant differences were found in the accuracy rates of Deepseek-R1 and ChatGPT-5.4 Thinking among the different question types (*p* > 0.05), indicating that each model demonstrated consistent performance across question types (Table 3).

**Table 3:** Comparison of accuracy rates across different question types [ *n* (%)].

Paper type	Question type	Deepseek-R1		ChatGPT-5.4Thinking	
		Correct	Wrong	Correct	Wrong
I Basic Knowledge (*n* = 972)	Clinical Knowledge	149 (92.55)	12 (7.45)	141 (87.58)	20 (12.42)
	Basic Theory	662 (93.50)	46 (6.50)	642 (90.68)	66 (9.32)
	Case Analysis	95 (92.23)	8 (7.77)	88 (85.44)	15 (14.56)
	*χ* ^2^	0.363		3.508	
	*P*	0.834		0.173	
II Related Professional Knowledge (*n* = 991)	Clinical Knowledge	232 (90.27)	25 (9.73)	226 (87.93)	31 (12.06)
	Basic Theory	561 (91.37)	53 (8.63)	527 (85.83)	87 (14.17)
	Case Analysis	110 (91.67)	10 (8.33)	109 (90.83)	11 (9.17)
	χ^2^	0.319		2.498	
	*P*	0.852		0.287	
III Professional Knowledge (*n* = 989)	Clinical Knowledge	253 (94.76)	14 (5.44)	238 (89.14)	29 (10.86)
	Basic Theory	499 (93.10)	37 (6.90)	481 (89.74)	55 (10.26)
	Case Analysis	166 (89.25)	20 (10.75)	169 (90.86)	17 (9.14)
	χ^2^	5.127		0.357	
	*P*	0.077		0.836	
IV Professional Practice Ability (*n* = 957)	Clinical Knowledge	254 (91.70)	23 (8.30)	244 (88.09)	33 (11.91)
	Basic Theory	440 (89.43)	52 (10.57)	432 (87.80)	60 (12.20)
	Case Analysis	175 (93.09)	13 (6.91)	172 (91.49)	16(8.51)
	χ^2^	2.547		1.936	
	*P*	0.28		0.38	

Data are presented as *n* (%). Percentages represent column proportions within each AI model. Question types were categorized as clinical knowledge, basic theory, and case analysis.

### 3.4 Analysis of AI model accuracy across different disciplines

Based on the favorable consistency observed across the three repeated test administrations, the results of the first test administration were used to further analyze the accuracy of the two models across five major disciplines (Clinical Laboratory Basics, Immunology, Microbiology, Biochemistry, and Hematology). The results showed that Deepseek-R1 exhibited no significant differences in accuracy across disciplines in any of the paper types (*p* > 0.05), whereas ChatGPT-5.4 Thinking showed significant differences in accuracy across disciplines in Papers I, II, and III (*p* < 0.05). Thus, Deepseek-R1 demonstrated more balanced performance across disciplines, while ChatGPT-5.4 Thinking exhibited a certain degree of disciplinary bias, with significant variations in accuracy across different subject areas (Table 4).

**Table 4:** Comparison of accuracy rates across different disciplines [*n* (%)].

Paper type	Discipline	Deepseek-R1		ChatGPT-5.4Thinking	
		Correct	Wrong	Correct	Wrong
I Basic Knowledge (*n* = 972)	Clinical Laboratory Basics	165 (90.16)	18 (9.84)	162 (88.52)	21 (11.48)
	Immunology	202 (91.82)	18 (8.18)	188 (85.45)	32 (14.55)
	Microbiology	162 (96.43)	8 (4.77)	156 (92.86)	14 (8.34)
	Biochemistry	213 (94.67)	12 (5.33)	212 (94.22)	13 (5.78)
	Hematology	164 (94.25)	10 (5.75)	153 (87.93)	21 (12.07)
	χ^2^	5.576		10.826	
	*P*	0.233		0.029	
II Related Professional Knowledge (*n* = 991)	Clinical Laboratory Basics	141 (87.58)	20 (12.42)	127 (78.88)	34 (21.12)
	Immunology	175 (91.15)	17 (8.85)	167 (86.98)	25 (13.02)
	Microbiology	197 (92.49)	16 (7.51)	194 (91.08)	19 (8.92)
	Biochemistry	224 (93.72)	15 (6.28)	218 (91.21)	21 (8.79)
	Hematology	166 (89.25)	20 (10.75)	156 (83.87)	30 (16.13)
	χ^2^	5.799		17.857	
	*P*	0.215		0.001	
III Professional Knowledge (*n* = 989)	Clinical Laboratory Basics	160 (90.91)	16 (9.09)	144 (81.82)	32 (18.18)
	Immunology	194 (94.63)	11 (5.37)	189 (92.20)	16 (7.80)
	Microbiology	181 (92.82)	14 (7.18)	181 (92.82)	14 (7.18)
	Biochemistry	221 (92.47)	18 (7.53)	220 (92.05)	19 (7.95)
	Hematology	162 (93.10)	12 (6.90)	154 (88.51)	20 (11.49)
	χ^2^	2.042		17.089	
	*P*	0.728		0.002	
IV Professional Practice Ability (*n* = 957)	Clinical Laboratory Basics	155 (90.64)	16 (9.36)	141 (82.46)	30 (17.54)
	Immunology	173 (90.58)	18 (9.42)	170 (89.00)	21 (11.00)
	Microbiology	202 (93.09)	15 (6.91)	197 (90.78)	20 (9.22)
	Biochemistry	207 (91.19)	20 (8.81)	204 (89.87)	23 (10.13)
	Hematology	132(87.42)	19(12.58)	136(90.07)	15(9.93)
	χ^2^	3.487		8.135	
	*P*	0.48		0.087	

Data are presented as *n* (%). Percentages represent column proportions within each AI model. Disciplines include Clinical Laboratory Basics, Immunology, Microbiology, Biochemistry, and Hematology.

### 3.5 Comparison of accuracy between the two AI models

Given the favorable consistency observed across the three repeated test administrations, the results from the first administration were used for analysis. A paired comparison of accuracy between the two models was performed across the four test papers to evaluate their overall performance difference on the junior professional qualification examination in medical laboratory technology. The results showed that Deepseek-R1 achieved significantly higher accuracy than ChatGPT-5.4 Thinking on Paper I (Basic Knowledge), Paper II (Related Professional Knowledge), and Paper III (Professional Knowledge) (*p* < 0.05). No significant difference was observed between the two models on Paper IV (Professional Practice Ability) (*p* > 0.05). These findings indicate that Deepseek-R1 outperformed ChatGPT-5.4 Thinking on test papers emphasizing theoretical knowledge, whereas the two models performed comparably on the paper emphasizing practical application ability (Table 5).

**Table 5:** Paired comparison of accuracy rates between the two AI models [*n* (%)].

Paper Type	Model	Comparison Result		Discordant pairs (D-C/C-D)	χ^2^	*P*
		Correct	Wrong			
I Basic Knowledge (*n* = 972)	Deepseek-R1	906 (93.21)	66 (6.79)	66/31	7.573	0.006
	ChatGPT-5.4 Thinking	871 (89.61)	101 (10.39)			
II Related Professional Knowledge (*n* = 991)	Deepseek-R1	903 (91.12)	88 (8.88)	68/27	8.28	0.004
	ChatGPT-5.4 Thinking	862 (86.98)	129 (13.02)			
III Professional Knowledge (*n* = 989)	Deepseek-R1	918 (92.82)	71 (7.18)	57/27	5.355	0.021
	ChatGPT-5.4 Thinking	888 (89.79)	101 (10.21)			
IV Professional Practice Ability (*n* = 957)	Deepseek-R1	869 (90.80)	88 (9.20)	48/27	2.263	0.132
	ChatGPT-5.4 Thinking	848 (88.61)	109(11.39)			

Data are presented as *n* (%). Percentages represent the proportion of correct or incorrect answers within each paper type for each model. “Correct” indicates that the model's answer matched the standard answer; “Incorrect” indicates a mismatch. Discordant pairs indicate the number of questions with discrepant answers between the two models. “D–C” denotes the number of questions where Deepseek-R1 was correct while ChatGPT-5.4 Thinking was incorrect; “C–D” denotes the number of questions where ChatGPT-5.4 Thinking was correct while Deepseek-R1 was incorrect.

### 3.6 Analysis of incorrect answer patterns

To elucidate the distribution of error patterns across question types and disciplines over three repeated test administrations, we performed a categorical analysis of incorrect answers. This is a descriptive analysis without statistical testing. For Papers I and IV, both AI models showed a similar pattern. The highest proportion of errors came from items answered incorrectly in all three administrations. Items with only one incorrect response out of three ranked second, while those with two incorrect responses accounted for the smallest proportion. On Paper II, both models also showed the highest proportion for items incorrect in all three administrations, followed by items with two incorrect responses, and the lowest proportion was items with one incorrect response. On Paper III, for Deepseek-R1, the highest proportion was items incorrect in all three administrations, followed by items with two incorrect responses, and the lowest was items with one incorrect response. For ChatGPT-5.4 Thinking on Paper III, the highest proportion was also items incorrect in all three administrations, followed by items with one incorrect response, and the lowest was items with two incorrect responses. Further analysis by question type and discipline showed a consistent pattern. For both models, items answered incorrectly in all three administrations consistently accounted for the highest proportion across all categories. These findings indicate that both AI models possess stable knowledge blind spots, characterized by persistent errors on specific questions rather than random fluctuations (Table 6).

**Table 6:** Distribution of error patterns across question types and disciplines.

Paper type	Model	Question type (*n*,%)					Discipline (*n*,%)				
		Count*	Total	Case Analysis	Basic Theory	Clinical Knowledge	Clinical Laboratory Basics	Immunology	Biochemistry	Microbiology	Hematology
I Basic Knowledge	Deepseek-R1	3	47 (55.49)	6 (12.76)	31 (65.96)	10 (21.28)	13 (52.00)	14 (70.00)	8 (50.00)	7 (58.33)	5 (41.67)
		2	15 (17.65)	1 (6.67)	11 (73.33)	3 (20.00)	3 (12.00)	5 (25.00)	3 (18.75)	1 (8.33)	3 (25.00)
		1	23 (27.06)	2 (8.70)	19 (82.61)	2 (8.69)	9 (36.00)	1 (5.00)	5 (31.25)	4 (33.33)	4 (33.33)
	ChatGPT-5.4 Thinking	3	76 (59.38)	11 (14.47)	51 (67.11)	14 (18.42)	14 (18.42)	25 (32.89)	11 (14.47)	10 (13.16)	16 (21.05)
		2	22 (17.19)	1 (4.55)	15 (68.18)	6 (27.27)	9 (40.91)	5 (22.73)	2 (9.09)	3 (13.64)	3 (13.64)
		1	30 (23.44)	3 (10.00)	21 (70.00)	6 (20.00)	12 (40.00)	5 (16.67)	5 (16.67)	4 (13.33)	4 (13.33)
II Related Professional Knowledge	Deepseek-R1	3	60 (54.05)	8 (13.33)	33 (55.00)	19 (31.67)	13 (21.67)	9 (15.00)	13 (21.67)	13 (21.67)	12 (20.00)
		2	28 (25.43)	2 (7.14)	20 (71.43)	6 (21.43)	8 (28.57)	7 (25.00)	3 (10.71)	3 (10.71)	7 (25.00)
		1	23 (20.72)	1 (4.35)	16 (69.57)	6 (26.09)	5 (21.74)	7 (30.43)	5 (21.74)	2 (8.70)	4 (17.39)
	ChatGPT-5.4 Thinking	3	89 (51.15)	7 (7.87)	59 (66.29)	23 (25.84)	17 (19.10)	23 (25.84)	14 (15.73)	15 (16.85)	20 (22.47)
		2	47 (27.01)	5 (10.64)	32 (68.09)	10 (21.28)	16 (34.04)	4 (8.51)	9 (19.15)	7 (14.89)	11 (23.40)
		1	38 (21.84)	6 (15.79)	23 (60.53)	9 (23.68)	18 (47.37)	4 (10.53)	5 (13.16)	2 (5.46)	9 (23.68)
III Professional Knowledge	Deepseek-R1	3	67 (65.05)	12 (17.91)	34 (50.75)	21 (31.34)	15 (22.39)	14 (20.90)	14 (20.90)	12 (17.91)	12 (17.91)
		2	20 (19.42)	1 (5.00)	18 (90.00)	1 (5.00)	3 (15.00)	6 (30.00)	4 (20.00)	1 (5.00)	6 (30.00)
		1	16 (15.53)	1 (6.25)	11 (68.75)	4 (25.00)	3 (18.75)	1 (6.25)	7 (43.75)	4 (25.00)	1 (6.25)
	ChatGPT-5.4 Thinking	3	87 (59.59)	15 (17.24)	44 (50.57)	28 (32.18)	24 (27.59)	15 (17.24)	19 (21.84)	17 (19.54)	12 (13.79)
		2	25 (17.12)	1 (4.00)	16 (64.00)	8 (32.00)	9 (36.00)	6 (24.00)	4 (16.00)	3 (12.00)	3 (12.00)
		1	34 (23.29)	4 (11.76)	23 (67.65)	7 (20.59)	5 (14.71)	11 (32.35)	5 (14.71)	6 (17.65)	7 (20.59)
IV Professional Practice Ability	Deepseek-R1	3	46 (49.46)	13 (28.26)	27 (58.70)	6 (13.04)	7 (15.42)	9 (19.57)	12 (26.09)	9 (19.57)	9 (19.57)
		2	19 (20.43)	3 (15.79)	11 (57.89)	5 (26.32)	8 (42.11)	3 (15.79)	4 (21.05)	2 (10.53)	2 (10.53)
		1	28 (30.11)	11 (39.29)	12 (42.86)	5 (17.86)	7 (25.00)	2 (7.14)	7 (25.00)	6 (21.43)	6 (21.43)
	ChatGPT-5.4 Thinking	3	76 (53.52)	15 (19.74)	41 (53.95)	20 (26.32)	22 (28.95)	10 (13.16)	17 (22.37)	10(13.16)	17(22.37)
		2	19(13.38)	3(15.79)	10(52.63)	6(31.58)	6(31.58)	3(15.79)	1(5.46)	7(36.84)	2(10.53)
		1	47(33.10)	3(6.38)	36(76.60)	8(17.02)	15(31.91)	7(14.89)	11(23.40)	4(8.51)	10(21.28)

Data are presented as *n* (%). Row percentages may not sum to 100% due to rounding. Error type: 3 = incorrect in all three administrations; 2 = incorrect in two administrations; 1 = incorrect in only one administration. The “Count” column indicates the number of questions with the corresponding error pattern.

### 3.7 Intern performance on the examination papers

**Figure 1:**
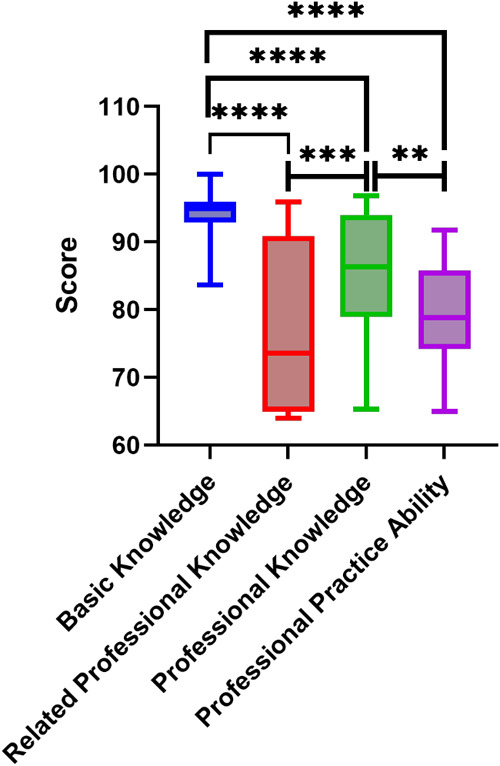
Distribution of accuracy scores among 46 interns. Each box represents the interquartile range (IQR, 25th–75th percentiles), with the horizontal line inside the box indicating the median. The whiskers extend to the minimum and maximum values within 1.5 times the IQR; no outliers were detected. The dashed horizontal line at 60% marks the official passing score. For each paper, the median, interquartile range (IQR, 25th–75th percentiles), range (min–max), and standard deviation (SD) are as follows:Paper I (Basic Knowledge): median 94.90%, IQR 92.86%–95.92%, range 83.67%–100.0%, SD 3.52; Paper II (Related Professional Knowledge): median 73.56%, IQR 64.95%–90.91%, range 63.92%–95.88%, SD 12.22; Paper III (Professional Knowledge): median 86.32%, IQR 78.95%–93.95%, range 65.26%–96.84%, SD 8.81; Paper IV (Professional Practice Ability): median 78.87%, IQR 74.23%–85.83%, range 64.95%–91.75%, SD 6.52.

The accuracy rates of the 46 interns across the four papers are presented in Figure 1. To evaluate the interns' clinical competency after their training, we administered the 2024 examination papers. The results showed that the interns performed best on Paper I (Basic Knowledge, *p* < 0.05) and worst on Paper II (Related Professional Knowledge, *p* < 0.05). Paper II also exhibited the largest dispersion (SD = 12.22).

## 4 Discussion

This study is the first to systematically evaluate two mainstream AI models, Deepseek-R1 and ChatGPT-5.4 Thinking, on a junior professional qualification examination in the highly specialized field of medical laboratory technology. By comparing their performance with that of clinical interns, this study reveals the potential and limitations of AI in professional knowledge assessment within this field. The results showed that both AI models achieved significantly higher overall accuracy than the interns. Deepseek-R1 outperformed ChatGPT-5.4 Thinking in the dimensions of basic knowledge, related professional knowledge, and professional knowledge, while the two models performed comparably in the dimension of professional practice ability. This study fills a gap in the systematic evaluation of AI in medical laboratory technology exams. It also provides important evidence for developing new educational tools, improving test preparation systems, and integrating AI more deeply into medical education.

### 4.1 Analysis of overall AI performance

This study found that both Deepseek-R1 and ChatGPT-5.4 Thinking demonstrated a high level of proficiency on the junior professional qualification examination in medical laboratory technology. Based on the overall performance across four test papers, Deepseek-R1 achieved significantly higher accuracy rates than ChatGPT-5.4 Thinking on Paper I, Paper II, and Paper III. In the Polish infectious disease specialty examination and the Chinese National Medical Licensing Examination, DeepSeek series models have shown performance comparable to or better than GPT series models (6, 16). In the emergency medicine specialty examination, although ChatGPT-4o achieved the highest overall accuracy, Claude 3.5 demonstrated superior response consistency (17). These studies indicate that the performance of different AI models varies across medical examinations, and the choice of model should be tailored to the specific application context.

The superior performance of Deepseek-R1 may be closely related to the composition of its training corpus. As a model natively developed in China, Deepseek-R1's training corpus contains a higher proportion of Chinese literature, textbooks, and specific knowledge such as Chinese healthcare policies and regulations (16). Although ChatGPT-5.4 Thinking supports multilingual input, its training environment remains predominantly English-centric, which may impose limitations when processing professional terminology and knowledge systems in a Chinese context. When the Chinese National Medical Licensing Examination questions were translated into English, GPT-4o's accuracy significantly increased from 75.3% to 83.5% (18). Wang et al. found that ChatGPT performed significantly better in English than in Chinese on the pharmacist licensing examination in Taiwan, China (19). Further observations by Alfertshofer et al. revealed substantial differences in ChatGPT's performance across medical licensing examinations in six countries, highlighting the influence of national and language factors (20). In the Chinese National Medical Licensing Examination, DeepSeek-R1 and GPT-4o performed comparably on Unit 3, whereas DeepSeek-R1 exhibited a distinct advantage on units involving culturally or linguistically specific content (16). These findings suggest that the advantages of linguistic environment and training corpus are more fully realized in knowledge-based content, whereas the performance gap between models narrows on more universal clinical practice questions (21, 22). Together, these results underscore the need for more in-depth research and optimization of generative AI models in multilingual and cross-cultural contexts to enhance their adaptability and accuracy across diverse scenarios.

### 4.2 Analysis of performance differences across question types and disciplines

This study analyzed the performance of two artificial intelligence models across different question types and subject areas. At the question type level, no statistically significant differences were observed in the accuracy rates of Deepseek-R1 and ChatGPT-5.4 Thinking across the three question types indicating that the two models exhibited comparable proficiency across different question formats. In the Polish infectious disease specialty examination, researchers found no significant difference in AI model performance between clinical case questions and non-case content (6); similar findings were reported in studies on the Chinese National Medical Licensing Examination (18). These observations suggest that current large language models demonstrate comparable capability in analyzing complex clinical cases and processing routine knowledge points, which carries positive implications for the potential application of AI in clinical practice.

At the disciplinary level, the two models showed distinct performance patterns. Deepseek-R1 exhibited no significant differences in accuracy across the five subject areas in all paper types, demonstrating good disciplinary balance. In contrast, ChatGPT-5.4 Thinking showed significant differences in accuracy across disciplines in Papers I, II, and III, indicating a certain degree of disciplinary bias. This discrepancy may be related to uneven disciplinary distribution in the model training data. Previous research has noted that ChatGPT exhibits performance variations across different medical systems, and students may strategically use AI models based on their strengths in specific subject areas (23). The findings of this study further corroborate this phenomenon and suggest that, in medical education applications, appropriate AI tools should be selected based on specific disciplinary requirements.

### 4.3 Analysis of AI response reproducibility/consistency

The high reproducibility of models is an important prerequisite for their reliable use in educational applications. Both models demonstrated excellent stability across three repeated test administrations, with Fleiss' Kappa coefficients exceeding 0.7, indicating that they provide highly consistent answers under standardized testing conditions. DeepSeek-R1 and GPT-4o both showed good temporal robustness in the Chinese National Medical Licensing Examination (24); in the emergency medicine specialty examination, the Claude 3.5 model exhibited the highest response consistency (17).

Despite generally favorable stability, we did observe a small number of items for which answers were not completely consistent across the three testing sessions. Subtle differences in prompt wording or contextual information may lead to significant deviations in generated content, potentially affecting their reliability in practical applications (2, 3, 25–27). A study by Kochanek et al. showed that the response consistency of GPT-4.0 for the same questions over a four-day period ranged from 85% to 88%, highlighting the impact of uncertainty in the content generation process (28). Incorrect information generated by AI can lead to serious consequences, including erroneous self-diagnosis, delays in medical care, dissemination of potentially harmful medical misinformation, and decreased public trust in healthcare professionals and institutions. For example, GPT once provided inaccurate descriptions of a high-sensitivity cardiac troponin point-of-care testing device approved by the U.S. Food and Drug Administration (FDA). Such “hallucination” responses are not isolated cases and underscore the limitations of generative AI models in ensuring information accuracy (4, 29, 30). In the medical field, high consistency of output is critical, and answer variability may pose significant risks. Therefore, further evaluation of the reproducibility and accuracy of ChatGPT in laboratory medicine is necessary to ensure its reliability as a reference tool for medical applications (31). These observations suggest that, although AI performs reliably in standardized test answering, its output is not entirely deterministic when processing complex or ambiguous information, and human verification remains necessary.

### 4.4 Comparison of AI models and interns across the four examination papers

All 46 interns scored above the official passing threshold of 60% on each paper, thereby meeting the requirements for the laboratory technologist certificate. However, their performance varied considerably across paper types. On Paper I (Basic Knowledge), the interns achieved a median accuracy of 94.90% (SD = 3.52), which was comparable to the three-run average of ChatGPT-5.4 Thinking (94.90%) and only slightly lower than that of Deepseek-R1 (approximately 97.28%). This finding indicates that the interns possessed a solid command of fundamental theoretical knowledge. On Papers II (Related Professional Knowledge), III (Professional Knowledge), and IV (Professional Practice Ability), the interns' median accuracies dropped to 73.56%, 86.32%, and 78.87%, respectively, with Paper II showing the largest dispersion (SD = 12.22). Notably, to ensure a fair comparison, the 46 interns and both AI models were evaluated using the exact same 2024 examination papers. Both AI models maintained high accuracy across these papers with minimal between-run variability. The largest performance gap between humans and AI emerged on Paper IV (Professional Practice Ability), where the interns' median was 78.87% vs. 95.44% for Deepseek-R1 and 87.37% for ChatGPT-5.4 Thinking. Thus, although the interns met the certification standard, their relative weakness lay in applying knowledge to clinically oriented questions rather than in basic factual recall.

A previous comparative study on comprehensive nursing examinations reported that students retained advantages in dimensions requiring practical experience, contextual judgment, and emotional understanding, whereas AI excelled in data processing and logical computation (32). The present findings are inconsistent with that pattern, as our interns performed worst on the practice-oriented paper. This discrepancy can be reasonably explained by differences in the nature of the two professions. Nursing education places strong emphasis on bedside communication, situational awareness, and direct patient interaction (33). These competencies can be cultivated through clinical rotations and are difficult for current large language models to replicate. In contrast, medical laboratory technology focuses more on standardised technical procedures, interpretative rules, and disease-specific biomarker patterns, which are more readily encoded in the training corpora of AI models. Furthermore, the interns in this study had completed only ten months of clinical rotations, a period that may be insufficient for developing robust applied reasoning skills in the laboratory context. Therefore, the divergence between our results and those of the nursing study likely reflects discipline-specific differences in the construct of “clinical practice ability” and the extent to which current AI systems can emulate the cognitive demands of each field.

### 4.5 Practical implications and future directions

The findings of this study have important practical implications for medical laboratory education. Both Deepseek-R1 and ChatGPT-5.4 Thinking can serve as efficient auxiliary tools for preparing for the laboratory technology qualification examination. Notably, Deepseek-R1, owing to its superior performance in the Chinese context and its free accessibility, is of particular value to students with limited resources. AI can be used to construct intelligent question banks and personalized learning systems. These systems dynamically deliver targeted exercises and knowledge explanations based on students' error patterns, enabling individualized instruction. Analysis of incorrectly answered items revealed that items consistently answered incorrectly across all three repeated administrations accounted for the highest proportion for both models. This finding suggests the presence of stable knowledge blind spots in the models, which may offer insights for optimizing teaching content. AI can also be integrated into instructional activities—for example, by simulating standardized case analyses and providing real-time feedback. Such integration helps compensate for the limitations of traditional teaching resources, enhances teaching efficiency, and strengthens students' clinical reasoning skills. In the application process, human verification and critical thinking regarding AI-generated outputs must be maintained.

### 4.6 Limitations

This study has several limitations. The questions used in this study were sourced from the junior professional qualification examination in medical laboratory technology administered between 2015 and 2024. All image-based questions were excluded because the number of such questions was very small and the black-and-white, low-resolution images in our electronic materials were unsuitable for reliable interpretation. This study only compared two mainstream models, Deepseek-R1 and ChatGPT-5.4 Thinking. Given the rapid development in the field of artificial intelligence, other competitive models, such as Claude and Gemini, exist; therefore, the conclusions of this study may not be fully generalizable to all AI models. Regarding stability assessment, this study employed three repeated test administrations, which meets the basic requirements for experimental research and provides preliminary validation of model stability. However, to further evaluate the long-term stability and potential random fluctuations of the models, future studies may consider designing repeated tests over a longer time span and with a greater number of iterations. Another important limitation is the risk of data contamination. The real examination questions used in this study are widely available in test-preparation books and online databases for the Chinese Medical Laboratory Professional Title Examination. Given that the knowledge cutoffs of Deepseek-R1 and ChatGPT-5.4 Thinking are later than the examination years, these questions are likely to have been present in the pre-training corpora of both models. The high accuracy rates observed may partially reflect memorization of training data rather than genuine reasoning ability. This is an inherent limitation shared by all studies that evaluate large language models using publicly available examination questions. To mitigate this issue, we disabled the web search function during testing; nevertheless, we cannot completely rule out the possibility that these real examination questions were included in the pre-training data. Readers should take this factor into account when interpreting the conclusions of this study. Consequently, any statements in this paper regarding the models' reasoning abilities should be considered preliminary and subject to confirmation using newly developed or unseen questions. This study used the official web-based chat interfaces rather than APIs. Consequently, we could not precisely control or freeze model versions, and the temperature parameter may not have been fully effective. Future studies using API access with fixed model snapshots and controlled parameters would provide stronger reproducibility. Regarding memory management, we took several precautions as described above. However, it should be noted that when using web-based chat interfaces rather than APIs, complete control over all memory-related parameters is not guaranteed. For example, some platform-level caching mechanisms may remain active despite our efforts. Future studies using API access with explicit stateless configurations would provide stronger guarantees against data contamination.

Regarding intern assessment, the 46 interns recruited in this study came from two universities, representing a relatively limited sample size that may introduce sampling bias. Future research could expand the sample size and adopt a multicenter recruitment strategy to enhance the representativeness of the findings. In addition, although this study systematically described the distribution of incorrectly answered items by question type and discipline, thereby identifying high-frequency error domains, it lacks in-depth qualitative analysis of the underlying mechanisms of these errors. This study was conducted in a static, time-unpressured, non-interactive environment, which differs from real examination settings and authentic learning processes. Consequently, the present results cannot simulate test-takers' performance under time pressure or the learning process in which students clarify questions and deepen understanding through multi-round dialogue with AI.

## 5 Conclusion

Both Deepseek-R1 and ChatGPT-5.4 Thinking performed well on the junior professional qualification examination in medical laboratory technology, showing high accuracy and consistency. Deepseek-R1 had an advantage in both accuracy and the detail of its explanations. Performance differences between the two models across disciplines suggest that their application should be tailored to specific contexts. Across the four test papers, both AI models achieved significantly higher accuracy rates than the interns. In medical laboratory education, AI should serve as an auxiliary tool. Combined with instructor guidance and active student thinking, it can support knowledge reinforcement, personalized self-assessment, and clinical reasoning training. Importantly, because all examination questions used in this study are publicly available historical papers that may have been included in the training corpora of both AI models, the high accuracy rates we observed may partly result from memorization rather than genuine reasoning. Therefore, our findings should be interpreted as evidence of strong performance on familiar question formats, not as definitive proof of reasoning ability. Future studies using newly created or unseen questions are needed to validate the models' true reasoning capabilities. This study is the first to systematically evaluate the application of artificial intelligence in the professional qualification examination for medical laboratory technology, providing important empirical evidence for the rational use of AI in education within this field.

## Data Availability

The original contributions presented in the study are included in the article/Supplementary Material, further inquiries can be directed to the corresponding author/s.
